# ‘I am your son, mother’: severe dementia and duties to visit parents who can’t recognise you

**DOI:** 10.1007/s11019-019-09931-5

**Published:** 2019-11-14

**Authors:** Bouke de Vries

**Affiliations:** Department of Historical, Philosophical and Religious Studies, Umeå University, Umeå, Sweden

**Keywords:** Dementia, Alzheimer, Memory loss, Filial duties, Loneliness, Parents, Adult children, Visits

## Abstract

It is commonly assumed that many, if not most, adult children have moral duties to visit their parents when they can do so at reasonable cost. However, whether such duties persist when the parents lose the ability to recognise their children, usually due to dementia, is more controversial. Over 40% of respondents in a public survey from the British Alzheimer’s Society said that it was “pointless” to keep up contact at this stage. Insofar as one cannot be morally required to do pointless things, this would suggest that children are *relieved* of any duties to visit their parents. In what appears to be the only scholarly treatment of this issue, Claudia Mills has defended this view, arguing that our duties to visit our parents require a type of relationship that is lost when parents no longer remember who their children are. This article challenges Mills’ argument. Not only can children be duty-bound to visit parents who have lost the ability to recognise them, I argue that many children do in fact have such duties. As I show, these duties are grounded in any special interests that their parents have in their company; the fact that visiting their parents might allow them to comply with generic duties of sociability; and/or the fact that such visits allow them to express any gratitude that they owe their parents.

## Introduction

One of the most tragic events in the lives of children is when one or both of their parents become incapable of remembering who they are. This is usually the result of severe dementia, but might have other causes as well, such as head injuries sustained in accidents (for the purposes of this article, I focus on cases where dementia is the cause). Although many children will continue to visit their parents in such cases—indeed the very idea of ceasing to do so might be inconceivable to them—some will stop providing (regular) company.^1^ Whilst the reasons for this might be manifold, a 2015 public survey by the British Alzheimer’s Society suggests that the belief that keeping up contact has become pointless will usually play a major role. In this survey, no less than 42% of respondents reported that, in their view, maintaining contact with a person who could no longer recognise them did not serve any purpose (BBC 2016).

My aim in this article is to investigate whether adult children who cease visiting their parents once their parents lose the ability to recognise them might be morally at fault. In what appears to be the only scholarly treatment of this issue, Mills (2003) has rejected this view. According to Mills, the kind of child-parent relationship that is necessary for children to have duties to visit their parents is *lost* when their parents become incapable of recognising them. After setting out her argument in more detail, I will take issue with this view. Not only can it be morally incumbent on children to visit parents who no longer remember who they are, my contention is that many children have such duties. As I show, these duties are grounded in any special interests that their parents have in their company (i.e. any interests that they have in their children’s company but not in most other people’s company with the possible exception of friends and other close relatives); the fact that visiting their parents might allow the children to comply with generic duties of sociability; and/or the fact that such visits allows them to express any gratitude that they owe their parents.

Before vindicating these claims, three clarificatory remarks are in order. First, my focus within this article is on parents who had both physical and legal custody of their children during the latter’s childhood, or a considerable part thereof. What this means that is that, during this period, the parents lived together with their children and were legally entitled to make important decisions about their upbringing, such as ones about their education, health care, and attendance of religious ceremonies. Whether children might have duties to visit parents who have not been closely involved within their lives—think of parents who were denied both types of custody early in their children’s lives, or those who put their children out for adoption shortly after they were born—is a question beyond this article’s remit.

Second, ‘visits’ are construed as actions whereby people move in space (or rather space–time) in order to provide at least one other person with their company. Children who visit parents with severe dementia often also provide them with physical care in situ (e.g. by helping them shower, get dressed, and take their medicine) and there are good grounds for thinking that, especially within ageing societies and societies with minimal welfare provisions, some children have a moral responsibility to do so. However, since I am only interested here in when, if ever, children have moral duties to visit their parents understood as duties to offer their *companionship* as opposed to physical support, I assume *arguendo* throughout this article that the parents’ physical needs are adequately met by care home staff, live-in care workers and/or robot carers even though this is frequently not the case in practice.

Third, when speaking of ‘parents who no longer recognise their children’, I am referring to individuals who are still conscious of their surroundings but who have lost the ability to identify their children as their own sons or daughters (in some cases, they might not even remember that they have children). Such cases ought to be distinguished from ones where parents have simply lost the ability to differentiate among their children insofar as they have more than one child, i.e. cases where they know that someone is their child but where they cannot recall which child.

## The conditionality view

Let us begin by looking at Mills’ view in more detail, which I will refer to as the ‘Conditionality View’ as it maintains that children’s moral duties to visit their parents are *conditional* upon their parents being able to recognise them. The reason why Mills believes that children are relieved of any duties to visit their parents when the parents lose the ability to recognise them is that the kind of relationship that she considers necessary for such duties to arise becomes impossible in such cases.

The obligation to participate in an ongoing relationship continues only when the relationship itself remains possible. I do not have—cannot have—an obligation to be in a *relationship* with someone who cannot be in a relationship with me. It is one of the tragedies of senility that genuine relationships with other human beings are no longer possible. ‘However, can’t I continue to love, unconditionally, someone who is senile and manifest this love to him or her in various ways, even if he or she is not able to recognize it?’ Yes. Ideally, familial love continues through all alterations, but, again, the relationship that is in many ways the foundation of the love cannot. Heartless as it may seem to say this, I see little point in spending extensive time with someone who does not know me for who I am. To do so is to engage in a pretence that a relationship still continues that, tragically, is gone forever (Mills 2003, p. 163).

In short, the reason why Mills thinks that children can no longer have duties to visit their parents when the parents lose the ability to recognise them is that, in her view, there is a “little point” in trying to maintain child-parent relationships when the capacity for such recognition is lost. Though she does not indicate *for whom* it is futile, one might reasonably expect that it includes the parents as filial duties are duties that are owed to parents.

What does this futility consist of? Whilst this too is not specified by Mills, there are two possible interpretations. The first is that the parents cease to derive *any* benefits from being visited by their children because, the thought goes, people can only benefit from being visited by individuals whom they recognise. Insofar as children can only have duties to do things for their parents that *benefit* their parents, it would follow from this that it is impossible for them to be duty-bound to visit their parents. The second interpretation accepts that parents (generally) have interests in being visited by their children even after losing the ability to recognise their children. However, it maintains that once they lose the ability to recognise them, they will lack any *special interests* in receiving filial visits, i.e. any interests over and above their interests in receiving visits from (equally socially skilled) strangers. To the extent that children can *only* have duties to visit their parents if their parents derive benefits from their visits that they do not derive from visits by most other individuals, it would follow from this that filial duties to visit cannot survive the loss of the parents’ capacity to remember who their children are either.

The next section suggests that the second interpretation of what the futility consists of is more plausible than the first. For now, it should be noted that insofar as parents cease to derive any special benefits from being visited by their children once they lose the ability to recognise them, this would not only relieve children from duties to visit their parents if we accept Mills’ relationship-account of filial duties (i.e. duties that children have towards their parents but not towards most other individuals)^2^ or other relationship-accounts that have been proposed by e.g. English (1992) and Jeske (2017). It would also relieve children of such duties if we accept another influential account of filial duties developed by Keller (2006). On this account, filial duties are generated by the fact that (many) children can provide their parents with goods that few, if any, other individuals can provide them with, such as the joy and wisdom that people might acquire from being closely involved within a person’s development throughout the course of her life and the feelings of intergenerational continuity and transcendence that interacting with one’s children might provide. Whilst filial duties can exist on this so-called ‘Special Goods Theory of Filial Duties’ *even when* children and their parents do not currently have a relationship, or simply not a particularly good one (cf. Keller 2006, p. 264), when parents cannot derive special benefits from filial visits due to their inability to recognise their children, their children cannot have duties to visit them based on this theory either.

## Against the conditionality view

My aim in this section is to challenge the Conditionality View. Its first interpretation, according to which children lack duties to visit parents who cannot recognise them *because* people cannot benefit from being visited by those whom they are unable to recognise, is most easily shown to be false. Whereas I will suggest shortly that such visits might serve several parental interests, my focus here is on hedonic or well-being interests. The reason for this is that, on most axiological theories,^3^ well-being—by which I mean the absence of pain and the presence of contentment and pleasure—is a morally significant good even if it is not the only thing that matters as some utilitarians contend.

When we focus on the hedonic effects of filial visits, it soon becomes clear that the first interpretation must be incorrect. Though it is sometimes frightening and distressing for people with advanced dementia to be visited by those whose identity is unknown to them—as it would be for many of us were a stranger to appear in our home—there is ample evidence that such visits can be, and frequently are enjoyable for these individuals (e.g. Lucero 2004, p. 174), as well as that the heighted feelings of well-being that they induce might persist for some time after the visit and even after the memory of the visit has been lost (Guzmán-Vélez et al. 2014).^4^ In fact, in many cases, the hedonic interests of people with severe dementia in being visited appear to be *stronger* than those of most other individuals. The reason for this is that this group is especially vulnerable to chronic loneliness,^5^ which has been found to contribute to a range of adverse outcomes, including depression (Cacioppo et al. 2010), poorer physical health (Aanes et al. 2010), and suicidal thinking (Stravynski and Boyer 2001).

One reason why people with (severe) dementia are particularly prone to chronic loneliness is to do with the social losses that older adults tend to experience. Not only does people’s social network shrink substantially as they reach old age,^6^ a large proportion of older adults loses their primary confidants due to the death of friends, partners/spouses, and siblings. Another reason is that compensating for these losses is often difficult for those with (severe) dementia. Apart from the fact that they might have age-related physical disabilities that reduce their ability to socialise, such as deafness and diminished mobility (cf. Moyle et al. 2011, p. 1449), their disease imposes various hurdles to maintaining existing relationships and forging new ones. Besides a progressively diminishing capacity to form memories and to retain existing memories, these might include an impaired ability to recognise faces,^7^ as well as various disease-related behaviours that make it less enjoyable for others to spend time with them, such as repeated questioning, shouting, and swearing.^8^ A final noteworthy hurdle concerns the stigma on dementia. Whilst this stigma has abated within many Western societies over the past decades (Hope 2010, p. 96), there continue to be reports of people being shunned by friends, relatives and other individuals upon revealing their dementia diagnosis (e.g. Moyle et al. 2011).^9^

### Special interests in filial visits

So far, I have suggested that the first interpretation of the Conditionality View must be false. But what about its second interpretation? As may be recalled, this interpretation concedes that many parents retain interests in being visited by their children after they lose the ability to recognise them. However, it maintains that, because of this loss, the parents lose any *special* interests in being visited by their children, i.e. any interests in being visited by their children rather than by (equally socially-skilled) strangers that derive from goods that their children can provide but most other individuals cannot, or simply not to the same degree. To the extent that parents need to have special interests in filial visits in order for their children to have duties to visit them, it follows on this view that children cannot be duty-bound to visit parents who have lost the ability to recognise them.

I believe that there are two problems with this argument. The first concerns its minor premise, according to which parents cease to have special interests in being visited by their children once they lose the ability to recognise them. As I argue below, even when this ability is lost, there are different ways in which filial visits might promote parental interests that visits by most other individuals cannot, or simply not to the same extent.

#### Well-being

To start with, some parents retain special interests in filial visits because their children are especially well-placed to promote their well-being, which I have defined as the absence of pain and the presence of contentment and pleasure. This will often be the case when their children are already visiting them on a regular basis. To see why, it should be noted that even when parents do not recognise them as their children, it is not uncommon for them to remember that they are the *same individuals* who visit them regularly, which may have the effect that they will miss them once they stop visiting or simply reduce the frequency of their visits (cf. Marley 2013).^10^

But that is not all; many children also have epistemic privileges that allow them to promote their parents’ well-being better than most other individuals with the possible exception of the parents’ romantic partners, siblings, friends, and a few other select individuals. To see this, it ought to be observed that, over the course of their lives, children tend to accumulate a considerable amount of information about their parents’ beliefs, preferences, and character traits, as well as about various episodes of the parents’ lives. This matters because such insider knowledge can be highly useful in deciding how to approach and interact with their parents so that their meet-ups become more enjoyable for the latter (and would expect in many cases themselves). For example, they might use this information to decide what topics to raise when talking to their parents; what kinds of gestures to make; when to be silent; and when to show affection and how. Especially when the parents have difficult personalities, getting these things right can have a big impact on their well-being.

#### Past autonomy

When people reach a level of cognitive impairment whereby they no longer recognise their children, they will have lost the capacity for autonomy understood as the ability to independently endorse a conception of the good life and live in accordance with it more or less successfully (cf. Colburn 2010). That does not necessarily mean, however, that they will have lost autonomy-interests in filial visits given that such visits might still honour their *past autonomy*. By this, I mean that such visits help to fulfil certain future-oriented preferences that the parents had when they were still autonomous, namely preferences to *continue to receive filial visits* if they were to lose the ability to recognise their children.

Though I am unaware of any survey data on this, such preferences appear to be widespread. Because of the love and affection that many parents have for their children, one may safely assume that a substantial share wants their children to remain part of their lives *even if* they were to lose the ability to recognise them. There might be exceptions to this; for example, some parents might not want to be visited by their children because they hold grievances against them, or because they do not want to be seen by their children in such an indigent state. Still another group may not wish to receive filial visits because of the burdens this places on their children or perhaps on other individuals who rely on their children’s care and support, such as their partners or own children. Nonetheless, especially when parents had minimally decent relationships with their children before they developed (severe) dementia and when their children do not live too far apart, one might expect that many would have wanted to continue to be visited by their children. When this is the case, filial visits fulfil a special parental interest given that *no one else* can honour these past autonomous preferences. (Of course, all this presupposes that individuals who have irretrievably lost the capacity for autonomy do indeed have interests in having their past autonomy honoured. Whereas any attempt to vindicate this view is beyond this article’s scope, I will just note here that other authors have offered compelling arguments for it,^11^ as well as that its moral significance is presupposed within many real-life contexts, including by the practice of letting advanced directives bear on decisions about end-of-life care and by the practice of allocating a diseased person’s property based on their final will.)

Thus far, I have suggested that for children to simply visit parents who have lost the capacity to identify them as their children might honour the parents’ past autonomy. Yet there are ways in which many children can interact with their parents during such visits that few others can that might honour *further* future-oriented preferences of the parents’ past autonomous selves. Prominent amongst these will be preferences to retain various memories for as long as possible. Such retention does not only matter to most people because they cherish specific memories (e.g. memories of their marriage, graduation, the birth of their children), but also because most of us regard memory-retention as a major part of identity-preservation, i.e. of remaining the same person.

Now although accessing memories becomes increasingly difficult for those who suffer from dementia, there is evidence that providing individuals with dementia with cues from their past can help many to access memories that would have otherwise remained inaccessible to them (Holton 2016).^12^ Such priming might occur, for instance, by showing them family pictures; playing music from their youth; or simply by recounting events from their lives. What is important for our purposes is that since children generally have greater knowledge about their parents’ lives than most other people and are more likely to have access to various personal objects from the parents’ lives, they will often be especially well-placed to provide such memory-inducing cues. When this is the case, the parents will have special interests in filial visits that are conditional upon their children helping them to remember their past during such visits.

At this point, some might say that retaining precious memories and preserving one’s identity through memory-retention are valuable *independently* of whether these things honour the preferences of our (past) autonomous selves. For example, in discussing the roles that friends and relatives can play in buttressing people’s identities through the provision of memory-inducing cues, authors such as Cowley (2018), Holton (2016), and Lindemann (2009) suggest that preserving one’s identity matters in its own right. Though I am sympathetic to such views, trying to vindicate them here would take us too far afield.

The same is true, *mutatis mutandis,* of views on which filial duties to visit parents who have lost the ability to recognise their children derive from the parents’ dignity interests. Such views are predicated on the assumption that even when parents no longer remember who their children are, it generally remains a greater affront to their dignity when their children refuse to visit them than when most other individuals refuse to do so, which is thought to be case *even when* the parents lack past autonomous wishes for their children to continue to visit them. Whilst I suspect that this is correct, a critic might argue that as long such parents receive enough social and emotional support from individuals other than their children, the notion that their dignity is undermined by their children’s refusal to (regularly) visit them simply reflects a social or cultural bias. (Which, of course, does not preclude the critic from holding the view that such dignity-harms might be incurred when the parents *do* recognise their children, as well as the view that there are ways in which children could harm the dignity of parents who no longer recognise them; all that she is committed to is that, under the conditions described, for children to refrain from visiting their parents does not cause such harm). Since a discussion of this topic is well outside this article’s purview, I will just note that, because dignity is such an abstract value,^13^ it is not clear whether defenders of the dignity-argument can answer our critic in a non-question-begging way. This is why despite being drawn to the conclusion of this argument, I do not wish to put much weight here on dignity-interests either.

### Generic duties of sociability

My criticism of the second interpretation of the Conditionality View has hitherto challenged the notion that when parents lose the ability to recognise their children, they cease to have any special interests in being visited by their children (i.e. any interests in being visited by them as opposed to strangers). Against this view, I argued that, in many cases, filial visits can still make contributions to the well-being and past autonomy of such parents that few, if any, other individuals can make. The aim of this subsection is to challenge the major premise of the second interpretation of the Conditionality View. According to this premise, children can *only* have duties to visit parents who cannot recognise them when, and because, their parents have special interests in being visited by them.

My objection to this premise proceeds on the assumption that we have what I call ‘generic duties of sociability’. These are duties to help ensure that others are provided with adequate opportunities for social contact when this can be done at reasonable cost,^14^ which are not just lacking when people are kept in isolation cells or in quarantine (to mention two extreme cases), but also when they are unable to visit other people as a result of severe dementia. What makes such duties generic is that they are owed to humanity as a whole, which distinguishes them from any duties of sociability that we have towards our friends and relatives specifically. Whilst the latter are based on our shared relationships, generic duties of sociability derive from the serious physical, psychological, and developmental harms that chronic loneliness has been found to engender (see the outset of this section). Such harms are not only problematic because they prevent lonely people from living minimally decent lives, although this is clearly a serious problem. They might also compromise people’s ability to provide (adequate) care to others, as well as adversely affect members of society more broadly when feelings of social exclusion give rise to anti-social behaviour (cf. Brownlee 2016a, b).

Whereas a lot more is to be said in defence of generic duties of sociability, I will not do so here as Collins (2013) and Brownlee (2013) have already offered elaborate and compelling defences. What is pertinent for us is that, inasmuch as we have generic duties of sociability, one may reasonably expect that the best way for many people to discharge these duties will involve providing company to any parents with severe dementia that they might have. There are at least two reasons for this.

The first is that individuals with (severe) dementia are especially vulnerable to chronic loneliness. As was noted, this is due to the social losses that people tend to experience in old age along with the distinct difficulties that those with dementia face in maintaining social contacts and forging new ones (see the outset of this section). Since any plausible account of generic duties of sociability will require people to make greater efforts to address the social needs of those who are at higher risk of (continued) loneliness than to address the social needs of those who are at lower risk, all other things being equal, it seems that the social needs of individuals with (severe) dementia will often need to be prioritised. (Notice that in this respect, generic duties of sociability are relevantly similar to moral duties to alleviate global poverty; all other things being equal, we have stronger reasons for giving money to those who are poorest and who need our resources the most than to those whose financial needs are weaker.) The second reason is that many children have a level of affection for their parents that they do not have for most other individuals with the possible exception of any partners, friends, and other close relatives that they might have. When such ties exist, it will typically be easier for people to establish or maintain a habit of visiting specific individuals, including those with severe dementia, compared to cases where these emotional connections are absent or simply not as strong. This is relevant because it means that by seeking to protect their parents from loneliness rather than individuals who are at equal risk of (continued) loneliness but towards whom they feel less affection, if any, children will be more likely comply with their generic duties of sociability, all other things being equal. (Notice that whilst most children will not reflect upon these issues and, consequently, not visit their parents with the *aim* of discharging generic duties of sociability, this does not undermine the point that I am trying to make, which is simply that generic duties of sociability can, and often will, give rise to duties to visit any parents with severe dementia that we might have.)

If the foregoing is correct, then even if parents generally ceased to have any special interests in being visited by their children once they lose the ability to recognise them (as I have denied within the previous subsection), it does not follow that their children cannot have duties to visit them. For as I have shown here, there are good grounds for thinking that we have generic duties of sociability and that for a large proportion of children, the most reliable way to discharge these duties involves visiting any parents with severe dementia that they might have.

### Gratitude

This brings us to a final possible ground of filial duties to visit parents who cannot recognise their children. Some will argue that children have duties to *show gratitude* towards their parents, at least when the parents have not done anything that would render displays of gratitude inappropriate such as physically abused their children (cf. Welch 2012), and that complying with these duties will often require that they (regularly) visit their parents even if the latter have lost the capacity to remember who they are.

Those who hold this view need to vindicate three assumptions. The first is that children can incur duties to show gratitude towards their parents as a result of the sacrifices that their parents have made for them and sometimes continue to make.^15^ The second assumption is that, for at least some children, such gratitude can only be duly displayed by protecting their parents from loneliness (among other possible ways in which they might have to express their gratitude that do not offer such protection, at least not in any straightforward sense; for example, they might be duty-bound to send packages to their parents with the latter’s favourite food or music.)^16^ The third assumption is that, for at least some of these children, protecting their parents from loneliness is possible *only* if they (regularly) visit them.

Can these assumptions be vindicated? Insofar as there are children who are duty-bound to show gratitude towards their parents, I think it is plausible that, in many cases, for them to discharge these duties will require that they help to protect their parents from loneliness, whatever else they might be required to do. This follows from the twin facts that (i) appropriate displays of gratitude will take into account the interests of the benefactors, and that (ii) many parents with severe dementia have stronger interests in being protected from loneliness than in receiving other goods from their children, including gifts. Given that (iii) a large share of children can only realistically protect their parents from loneliness by visiting them—even when there is the possibility of paying others to provide (regular) company on their behalf, doing so will be prohibitively expensively for many, apart from the fact that it might contravene the parents’ past autonomous preferences (see the penultimate subsection)—this would suggest that many children with parents who cannot recognise them have gratitude-based duties to visit their parents.

If I am right that (i)–(iii) are correct, then the success of gratitude-based arguments for filial duties to visit parents who have lost the ability to recognise their children turns on whether it is possible for children to owe their parents displays of gratitude. Whereas several authors subscribe to this view (e.g. Berger 1975; Blustein 1982; Wicclair 1990), it should be noted that it is not uncontroversial. For example, Wellman (1999) has argued that gratitude is a virtue and that this precludes it from being something that can be owed. Yet even when duties of gratitude are not considered to be incoherent, my experience is that a significant proportion of scholars believes that filial duties of gratitude are rare.^17^ One common reason for this is that they believe that only extraordinary sacrifices—whether supererogatory or not—can give rise to duties of gratitude, and that most parents do not make such sacrifices for their children. Another is that they think that we can only owe gratitude for acts to which we have meaningfully consented. Since many of the sacrifices that parents make for their children are ones to which the children never (meaningfully) consent as they are made during the latter’s (early) childhood, this too would substantially reduce the scope of filial duties of gratitude.

I believe that there are some promising responses to these objections (see e.g. Jeske 2017, pp. 373–375; and Schinkel 2012, pp. 400–403). As a discussion of these is beyond this article’s remit, however, I will just end by noting that if one believes that at least some children have duties to show gratitude towards their parents, then it is *even more likely* than I have suggested here that children can, and oftentimes do, have duties to visit parents who no longer recognise them. In an age where many societies are ageing rapidly and where a cure for Alzheimer and other forms of dementia remains to be found, these are important conclusions.

## Footnotes

1 Compare Lucero (2004, p. 174).

2 Notice that such duties are different from those that are owed to all human beings, such as duties not to torture people. At the same time, they do not require that the things that children owe their parents are owed *exclusively* to their parents. For example, insofar as we have duties to show gratitude to those who have made significant sacrifices for us (more on this within the final section), then this might not just generate duties to show gratitude towards our parents, but also towards a select number of other individuals who have made such sacrifices, such as friends who have supported us through thick and thin.

3 I.e. theories about what kinds of things are non-instrumentally valuable.

4 It is important though that people with dementia be approached the right way in order for these positive effects to occur. This means, *inter alia,* that their visitors should avoid a number of things, including using long sentences; asking many open-ended questions; asking people with dementia directly whether they remember various events; revealing their frustrations; and using Elderspeak (Alzheimer’s Society 2017).

5 Loneliness consists of a negatively experienced mismatch between one’s desired and realised social contact. See e.g. de Jong-Gierveld (1987).

6 See, for instance, Lang and Carstensen (1994).

7 For a discussion of the causes of facial recognition problems among people with dementia, see Lavallée et al. (2016).

8 Compare Hope (2010, p. 63).

9 To the extent that negative stereotypes about dementia are internalised, moreover, there is a risk that this will undermine the willingness of people with dementia to socialise as they come to feel unworthy of others’ company; cf. Hope (2010, p. 98).

10 For a more elaborate discussion of the importance of stable companionship to people’s well-being, see Brownlee (Brownlee 2016b, pp. 44–45).

11 See, for instance, Dworkin (1994) and Porteri (2018). In Dworkin’s view, the past autonomy-interests of individuals with severe dementia might even trump their current well-being interests in remaining alive. Specifically, he believes that any advanced directives that people have signed to be denied life-saving medical treatment if they develop severe dementia should be respected *even if* they live perfectly happy lives once they reach this stage of cognitive impairment.

12 The main exception are those who suffer from semantic dementia, who account for circa 2 percent of all people with dementia (Rogers and Friedman (2008).

13 As exemplified by the fact that it is often invoked by both sides in arguments. For further discussion, see Ashcroft (2005).

14 Without trying to list all possible scenarios where such costs are unreasonable, I take it that this will be the case when helping to provide others with opportunities for social contact makes it more difficult for people to make a living or to look after the physical needs of those for whose care they are responsible.

15 Two comments on filial duties to show gratitude towards parents are in order. First, whereas on some accounts, such duties can arise only when parents have done *more* for their children than they were morally required to, this need not be the case. For example, Schinkel (2012, p. 402) has argued that non-supererogatory parental investments might generate filial duties to show gratitude as long as the investments were sufficiently costly for the parents to make and were made out of genuine concern for the children’s well-being. Second, discharging such duties does not necessarily require that children *feel grateful* towards their parents. Those who reject such a requirement will usually do so because they believe that humans have insufficient control over their emotions in order to expect anything of the sort. Of course, this does not preclude them from accepting that there can be duties to *try to* feel grateful towards one’s parents, for example by thinking actively about all the things that one’s parents have done for one.

16 I am indebted to an anonymous reviewer for suggesting alternative ways of expressing gratitude to people with severe dementia.

17 This is based on personal communication at various academic events. I am not aware of scholars who have defended this view in print. .
